# Supplementary material for: A novel bivalent chromatin associates with rapid induction of camalexin biosynthesis genes in response to a pathogen signal in Arabidopsis
Source: eLife. 2021 Sep 15;10:e69508. doi: 10.7554/eLife.69508 (PMC8547951; doi:10.7554/eLife.69508)
Supplement: Supplementary file 2. [file elife-69508-supp2.docx]

Supplementary File 2. Primers used to quantify the abundance of H3K27me3 and H3K18ac in the genomic regions of camalexin biosynthesis genes using ChIP-qPCR in wild type and mutant plants with or without FLG22 treatment.

| Gene | Primer | Sequence |
| --- | --- | --- |
| CYP79B2 | P1-F | GACTTAAGACTTAGTCTCGGT |
| CYP79B2 | P1-R | TAGCCATTATGTTTTGGGTACG |
| CYP79B2 | P2-F | CAATGTATACGTTACAGAT |
| CYP79B2 | P2-R | TTATGTCTAGCTAGTATTG |
| CYP79B2 | P3-F | CGAAGTATTAGATCAATGT |
| CYP79B2 | P3-R | TCCTCGTGTTATATATGCAC |
| CYP79B2 | P4-F | GGCAGGTCACCAACAAAAC |
| CYP79B2 | P4-R | GTAGCATCACTAAGGTTATAG |
| CYP79B2 | P5-F | CAAGAAATTGATGACGGATC |
| CYP79B2 | P5-R | AGAGAGGATCTTCTGAGCG |
| CYP79B2 | P6-F | GCGTGATCACTCCCTTTGGT |
| CYP79B2 | P6-R | TCCACCGTCAGGTGCAGTG |
| CYP79B2 | P7-F | CCCACCGTAGAAGATGTAGA |
| CYP79B2 | P7-R | TGCCTTGTTCGTCTTTGAT |
| CYP79B2 | P8-F | GCTTACCGCCGATGAAATCA |
| CYP79B2 | P8-R | TGACTTCCTTTAGGGATGTG |
| CYP71A13 | P1-F | GTTCATCATCACTAGTCTTAC |
| CYP71A13 | P1-R | CATAAGTCTTAAGATCGACG |
| CYP71A13 | P2-F | GCAGGATTTACTGAGTTAAAG |
| CYP71A13 | P2-R | GGAATTAAACGTAATCTTTC |
| CYP71A13 | P3-F | CTACTATCATAGGTCGGCT |
| CYP71A13 | P3-R | GGAAGAGTTTATTAGCGACA |
| CYP71A13 | P4-F | TAGCATGCAGAATATGAGT |
| CYP71A13 | P4-R | GAACGGTGAGGATGGAGGC |
| CYP71A13 | P5-F | CTCAGTCTCAGGTACGGAC |
| CYP71A13 | P5-R | CACTCTTCATCTGTCTCCAG |
| CYP71A13 | P6-F | TGGTTGAATCCTTTGAGAAG |
| CYP71A13 | P6-R | GCCTCACTCGCTTCTTGAG |
| CYP71A13 | P7-F | CATATTGGCATGGATAGATG |
| CYP71A13 | P7-R | GTAGAGTCGAAGTTGTTGACG |
| CYP71A13 | P8-F | GGACGATGACGGAACTGATC |
| CYP71A13 | P8-R | GCAGTGTCTCGTTGGATCG |
| PAD3 | P1-F | CACCGCTAAAATTGTTGAC |
| PAD3 | P1-R | CATGTTTATCCTTAATGAGC |
| PAD3 | P2-F | GGATTTGTAATCTACTTTC |
| PAD3 | P2-R | CAGTATTGGGTCGTCTCAAGC |
| PAD3 | P3-F | CGGTCAGTGAAGTCTACAT |
| PAD3 | P3-R | GAATAATAATCGTAAGTGGAC |
| PAD3 | P4-F | CAAGCTACAGCGGATAGTAG |
| PAD3 | P4-R | TTGGACCCGGAGGAAGCTTA |
| PAD3 | P5-F | AGAAGCTTCCCATCATCGG |
| PAD3 | P5-R | CATCCCGATGTCTTTGAAG |
| PAD3 | P6-F | CGGTGACGAGTGGAGTCTGA |
| PAD3 | P6-R | TCCACGACTCTATCAGCTTC |
| PAD3 | P7-F | GTGCTAAAGGCTGAAGCGGT |
| PAD3 | P7-R | GTGGTGAACTTGAGAGCATC |
| PAD3 | P8-F | CGGACATATTTGTAGCAGG |
| PAD3 | P8-R | AAGAGTGGAGTTGTTGGATG |
