# Supplementary material for: A novel bivalent chromatin associates with rapid induction of camalexin biosynthesis genes in response to a pathogen signal in Arabidopsis
Source: eLife. 2021 Sep 15;10:e69508. doi: 10.7554/eLife.69508 (PMC8547951; doi:10.7554/eLife.69508)
Supplement: Supplementary file 3. [file elife-69508-supp3.docx]

Supplementary File 3. Primers used in the sequential ChIP-qPCR experiment to examine the co-localization of H3K27me3 and H3K18ac within camalexin biosynthesis genes.

| Name | Sequence |
| --- | --- |
| CYP79B2_qF | GCAATGGAAGAGATCGACAGAG |
| CYP79B2_qR | GGAGGATAGCTTTGACGTAGTTTAG |
| CYP71A13_qF | CCAACGAGACACTGCGATATG |
| CYP71A13_qR | GATCCGAATGGGATGTAGTTCAG |
| PAD3_qF | GCAGCAGAGGAAGTGCTAAA |
| PAD3_qR | ATCCCGATGTCTTTGAAGTTGT |
| GLK1_qF | GATTTAGAGCACCGCCAGTT |
| GLK1_qR | GAGCACCACCAAATCCAAGA |
| H3K27me3_only_qF | CAACGGTTCTTCATCCGATT |
| H3K27me3_only_qR | CTGCTCGAAATGGCTCTACC |
| H3K18ac_only_qF | GGTAACCGATGTGGGACATTT |
| H3K18ac_only_qR | CCAACAAGACTGGTCCAAAGA |
| Negative_con_qF | TGCTCGTCCCATTTCCTATC |
| Negative_con_qR | GGCATAGTGATTTTGCCACA |
