# Supplementary material for: A novel bivalent chromatin associates with rapid induction of camalexin biosynthesis genes in response to a pathogen signal in Arabidopsis
Source: eLife. 2021 Sep 15;10:e69508. doi: 10.7554/eLife.69508 (PMC8547951; doi:10.7554/eLife.69508)
Supplement: Supplementary file 4. [file elife-69508-supp4.docx]

Supplementary File 4. Primers used to examine the expression of camalexin biosynthesis genes under FLG22 induction using qPCR.

| Name | Sequence |
| --- | --- |
| CYP79B2_qF | ATCTCCTCTCAACACTTCAAGC |
| CYP79B2_qR | GGTGGCAGATACGGTTTCTTT |
| CYP71A13_qF | CGATTTGACTGGAGGGTAGAG |
| CYP71A13_qR | CCGAAGATGGAAATGCAATGAG |
| PAD3_qF | GCAGCAGAGGAAGTGCTAAA |
| PAD3_qR | ATCCCGATGTCTTTGAAGTTGT |
