# Supplementary material for: A novel bivalent chromatin associates with rapid induction of camalexin biosynthesis genes in response to a pathogen signal in Arabidopsis
Source: eLife. 2021 Sep 15;10:e69508. doi: 10.7554/eLife.69508 (PMC8547951; doi:10.7554/eLife.69508)
Supplement: Supplementary file 5. [file elife-69508-supp5.docx]

Supplementary File 5. The effect of genotype, FLG22 treatment, and time point on the expression change of camalexin genes.

|  | CYP79B2 | | | CYP71A13 | | | PAD3 | | | |
| --- | --- | --- | --- | --- | --- | --- | --- | --- | --- | --- |
| Effect | DF | p value | p<.05 | DF | p value | p<.05 | DF | p value | p<.05 |  |
| Genotype | 4 | 6.96E-08 | * | 4 | 3.40E-11 | * | 4 | 1.92E-07 | * |  |
| Treatment | 1 | 1.26E-24 | * | 1 | 2.02E-29 | * | 1 | 1.48E-36 | * |  |
| Time | 4 | 9.30E-04 | * | 4 | 3.21E-54 | * | 4 | 1.61E-38 | * |  |
| Genotype: Treatment | 4 | 4.74E-11 | * | 4 | 7.88E-08 | * | 4 | 9.80E-11 | * |  |
| Genotype: Time | 16 | 3.48E-08 | * | 16 | 7.99E-15 | * | 16 | 1.50E-06 | * |  |
| Treatment: Time | 4 | 4.86E-06 | * | 4 | 2.95E-42 | * | 4 | 2.14E-32 | * |  |
| Genotype: Treatment: Time | 16 | 1.59E-01 |  | 16 | 1.55E-12 | * | 16 | 3.90E-07 | * |  |
| Trial | 2 | 6.43E-01 |  | 2 | 3.12E-01 |  | 2 | 4.35E-01 |  |  |
