# Supplementary material for: A novel bivalent chromatin associates with rapid induction of camalexin biosynthesis genes in response to a pathogen signal in Arabidopsis
Source: eLife. 2021 Sep 15;10:e69508. doi: 10.7554/eLife.69508 (PMC8547951; doi:10.7554/eLife.69508)
Supplement: Supplementary file 6. [file elife-69508-supp6.docx]

Supplementary File 6. The effect of genotype, FLG22 treatment, and time on camalexin accumulation.

| Effect | DF | p value | p<.05 |
| --- | --- | --- | --- |
| Genotype | 4 | 1.57E-05 | *** |
| Time | 4 | <2.00E-16 | *** |
| Treatment | 1 | 7.71E-09 | *** |
| Genotype: Time | 16 | 2.13E-01 |  |
| Genotype: Treatment | 4 | 6.20E-02 |  |
| Time: Treatment | 4 | 9.48E-10 | *** |
| Genotype: Treatment: Time | 16 | 9.50E-01 |  |
| Trial | 1 | 5.85E-01 |  |
